# Understanding the sexual and reproductive health needs of immigrant adolescents in Canada: A qualitative study

**DOI:** 10.3389/frph.2022.940979

**Published:** 2022-07-22

**Authors:** Salima Meherali, Samantha Louie-Poon, Sobia Idrees, Samar Kauser, Shannon Scott, Bukola Salami, Helen Valliantos, Kainat Moez Meherali, Krishan Patel, Puja Suthar, Zohra Akbarzada, Ivan Marcus, Manjot Khangura, Abneet Mangat

**Affiliations:** Faculty of Nursing, University of Alberta, Edmonton, AB, Canada

**Keywords:** immigrant, adolescent, sexual and reproductive health, information needs, Canada

## Abstract

**Background:**

Literature suggests that immigrant adolescents receive limited sexual and reproductive health (SRH) education and rarely utilize SRH services in Canada. This study sought to explore the SRH information needs of immigrant adolescents in the province of Alberta.

**Methods:**

A qualitative descriptive methodology was undertaken to conduct 21 individual interviews with immigrant adolescents in Alberta.

**Results:**

A total of four themes emerged from the interviews: (1) Barriers to SRH; (2) needs of adolescents regarding SRH; (3) sources of knowledge; and (4) strategies to improve SRH. Our findings document the conflicting needs and preferences between adolescents and their parents regarding access to SRH resources and services.

**Discussion:**

Adolescents often felt unprepared to deal with their SRH issues due to socio-cultural barriers and conflicts with their parents' conservative attitude toward SRH concerns. Structural barriers to accessing SRH resources and services were also reported, including the location and cost of services. As a result, the majority of adolescents relied on digital methods to receive SRH information.

**Conclusion:**

This study highlights that future research and SRH service provider efforts need to remain cognizant of the positionality of immigrant adolescents and explore innovative ways to deliver SRH resources and services that meet their unique needs.

## Background

Inclusion and diversity are celebrated as pillars within Canadian society. As a welcoming place, Canada is home to a large immigrant population, with over 300,000 immigrants that arrived in 2016, the largest number since 1971 (1). Among this population, young people make up a significant portion of immigrants in Canada (2). The World Health Organization (2016) categorizes adolescents as young people between the ages 10 and 19 years (3). During this time, adolescents experience many biological changes, have an increased sense of maturity, and become curious about human relationships (4–6). Importantly, this developmental phase, marked by physiological and emotional change, is a critical time to establish and adopt lifelong healthy behaviors (4, 7).

For immigrant adolescents, specific needs are warranted to achieve healthy behaviors that adequately navigate their cultural identities, migration contexts, family expectations, and emerging attitudes developed within the Canadian context (8). For example, in some cultures of immigrant populations, sensitive topics such as sexual and reproductive health (SRH) are rarely discussed during adolescence. In many cases, exploring SRH during adolescence is considered taboo and unacceptable due to existing cultural and religious standards developed in pre-migration contexts (9, 10). Without resources and services that attend to the unique needs of immigrant adolescents, topics such as SRH may become further stigmatized as adolescents continue into adulthood.

## Introduction

The right to sexual health and well-being exists for every individual; this can be achieved by providing accessible and comprehensive sexual education and services, educating about the risks involved, and educating about the adverse consequences of unprotected sexual activity (11). According to World Health Organization, if adolescents initiate sexual activities without having adequate knowledge and skills regarding sexual protection lead them at higher risk of unwanted pregnancies, sexually transmitted infections including HIV/AIDS and unsafe abortions (11). A growing body of literature has documented that immigrant adolescents receive limited SRH education and rarely utilize SRH services in Canada (12–14). Research has also identified that there are significant gaps in the SRH knowledge of immigrant adolescents in Canada, including fertility and contraception, unintended pregnancy, HIV/STI transmission and protection, and menstruation (9, 10, 12, 15, 16). Additionally, literature reports that immigrant adolescents lack awareness and knowledge of Canadian confidentiality policies of services providers and how to navigate Canadian sexual health services (13, 17). These knowledge and service gaps are concerning given that immigrant adolescents may have difficulty in navigating and identifying their unique SRH needs within the Canadian context, which may bring about internal conflicts given the stark differences when compared to their pre-migration context.

To attain Canadian promises of inclusion and diversity, developing SRH resources and services specific to the adolescent immigrant population is paramount. In the absence of SRH resources and services that are inclusive of immigrant adolescents' needs, the young immigrant population in Canada may adopt unhealthy SRH attitudes and behaviors or experience exclusion and discrimination within SRH care (8). Despite these concerns, there is a lack of research on adolescent SRH needs from the perspective of immigrant adolescents within the Canadian context. To address this gap, our study sought to explore the SRH information needs of immigrant adolescents in the province of Alberta, a major destination for immigrant populations in Canada. This research study was done to answer the following research questions:

- What are the SRH information needs of adolescent immigrants in Canada?
- What are the strategies that can improve access to SRH services among adolescents immigrant in Canada?

## Methods

The current study was part of a larger community-based research study examining the sexual and reproductive health needs and rights (SRHR) of immigrant adolescents in Canada consisting of four phases: (1) development of an adolescent advisory group; (2) scoping review of the literature; (3) qualitative interviews; (4) adolescent advisory group engagement evaluation. This paper reports phase 3 of the larger study utilizing qualitative descriptive methodology (18).

### Data source

The data was collected from 21 adolescent immigrants through interviews in 2021. SLP and SK followed up with interested participants by email to schedule a virtual interview at a mutually agreed-upon date and time.

### Sampling

Purposive sampling was employed for participant recruitment using two strategies (19, 20). First, study information was posted to the social media accounts (Facebook, Instagram) of our research team member (SK) using a recruitment graphic that included the study purpose, eligibility criteria, and the research team's contact information. Second, the same study information was disseminated to the University of Alberta's Undergraduate Student Digest weekly email.

### Eligibility criteria

Participants were recruited in the study by following the eligibility criteria: (a) immigrant adolescent; (b) aged 14 and 19 years; (c) fluent in English; and (d) living in Alberta, Canada.

### Data collection

Data collection and analysis occurred iteratively until data saturation was achieved (20). After written informed consent was obtained, participants' demographic information was collected. Two research team members from Alberta (SLP, SK) trained in qualitative data collection methods conducted a total of 21 individual interviews. The decision to end data collection was an ongoing topic of discussion within the research team and based on the processes of data analysis. Specifically, the occurrence of redundancy within the themes, and rich substantiation suggested that data collection could be stopped (21). A semi-structured interview guide was used to explore the SRH information needs and experiences of immigrant adolescents (see Supplementary Document A). The interviews took place virtually on Google Meet using a secure and unique invitation link. Individual interviews lasted between 20 and 45 min. During seven interviews, one participant from the research team's adolescent advisory group was present. For the purposes of enhancing the larger project, the adolescent advisory member provided their input in the interview guide and observed the interview process to provide the research team with insights about the larger study during the monthly adolescent advisory group meeting. The advisory group members signed a confidentiality agreement form prior to observing the interviews. Our reporting of this study follows the Consolidated Criteria for Reporting Qualitative Research checklist (22).

### Data analysis

All interviews were digitally recorded, professionally transcribed verbatim, and cleaned for accuracy and completeness. Transcripts were uploaded to NVivo data management system. Inductive thematic data analysis was performed by two research team members (SLP, SK) trained in qualitative data analysis methods (23). First, all transcripts were read in detail several times (SLP, SK). Second, SLP and SK conducted open coding of all transcripts and grouped codes into preliminary categories. Research team members (SLP, SM, SK) reviewed and discussed codes and preliminary categories. Third, preliminary categories were grouped into a framework according to recurring themes pertaining to participants' experiences and information needs. All authors discussed and reviewed themes. Demographic data were analyzed using descriptive statistics. To ensure rigor SLP and SK kept a comprehensive audit trail and field notes throughout the data collection and analysis phases to document all decisions, modifications, and impressions. We also shared the transcribed data with four participants to ascertain whether the transcribed data accurately reflected their contributions Preliminary findings, interpretations, and research processes were continuously reviewed and discussed between the research team (SLP, SM, SK, SI).

### Ethics approval

Ethics approval was obtained from the University of Alberta Health Research Ethics Board (Pro00097730).

## Findings

A total of 21 adolescents participated in the study. The demographic data of the participants are reported in Table 1. Participants' parents' demographic characteristics are available in Supplementary File B. In this section, the word “adolescents” is interchangeably used with the “study participants” or “participants”.

**Table 1:** Demographic characteristics of the participants.

**Characteristics**	**Number (** ***f*** **)**	**Percentages (%)**
**Age (in years)**		
14–15 years	1	4.76
16–17 years	2	9.52
18–19 years	18	85.72
**Gender**		
Male	1	4.77
Female	20	95.23
**Education**		
Post-secondary	19	90.48
Grade-9	1	4.76
Grade-11	1	4.76
**Length of Stay in Canada**		
>10 years	13	61.90
4–9 years	7	33.40
1–3 years	1	4.77
**Primary Language of Adolescents**		
English	2	9.50
English and Hindi	2	9.50
Russian	1	4.76
English and Yoruba	1	4.76
English and Tagalog	2	9.50
English and Malayalam	1	4.76
English and Bengali	3	14.44
English and Spanish	2	9.50
English and Nepali	1	4.76
English, Tamil and Hindi	2	9.50
English and mandarin	1	4.76
English and Gujrati	2	9.50
English, Hindi, Urdu, and Punjabi	1	4.76
**Participants' Country of Birth**		
India	6	28.70
Philippine	2	9.50
Bangladesh	2	9.50
China	1	4.76
Bahrain	1	4.76
Nigeria	1	4.76
Nepal	1	4.76
Venezuela	1	4.76
Columbia	1	4.76
Saudi Arabia	1	4.76
Malaysia	1	4.76
Kuwait	1	4.76
Ireland	1	4.76
Kazakhstan	1	4.76

## Findings from the participants' interviews

**Figure 1:**
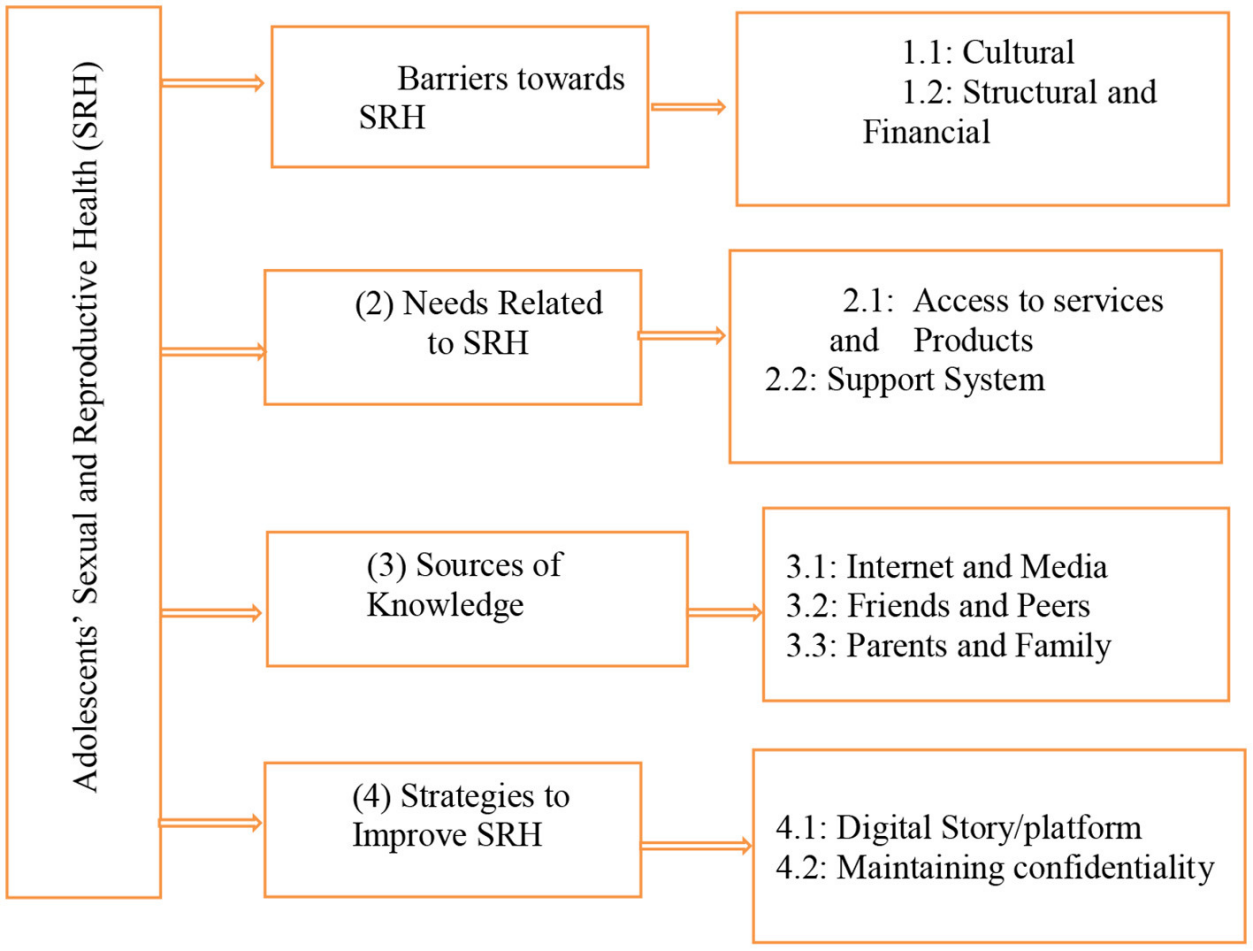
Thematic framework on findings from adolescents' interviews.

A total of four themes were identified; (1) barriers toward SRH, (2) needs of adolescents regarding SRH, (3) sources of knowledge, (4) strategies to improve SRH. These themes emerged from the nine sub-themes as presented in Figure 1.

### Barriers toward SRH

The first theme of the study focused on the barriers for adolescents in accessing products and services related to SRH. The most commonly reported types of barriers include cultural, structural, and financial.

#### Cultural

The participants shared that they had challenges due to cultural influence and conflicts with their parents' preferences and their choices in dealing with their SRH. For example, one participant said that, “my mom and all of our relatives don't use tampons, but I really wanted to get into that and start using them” (P. 1). Likewise, one participant shared that, “...sometimes I have concerns about myself, so I want to go and visit a doctor, but my parents are immigrants, it's been a little bit tough because they feel uncomfortable with the topic” (P. 1).

Other participants shared that if they prefer to go to a physician or a consultant, they keep it a secret from their parents. As one participant shared, “like if I have to go to a doctor about something, but I don't want to tell my parents” (P.2). Most of the participants expressed that they often found it challenging to openly communicate with their parents regarding their SRH needs and preferences. For example, one of the participants said;

it's sometimes hard to talk about this stuff, I have to talk about it with friends if I need to. It makes me a little uncomfortable if I talk about it with my family and my parents. Like it just feels weird (P. 1).

Another participant shared the similar concerns;

Oh. I cannot really talk about it with my dad [Laughs], because I guess there are like two sides to this rejection to talk to him about it. Because on my side, it's apparently because I'm embarrassed. I'm not saying I don't trust him, but I just – honestly, I think he might have some irrelevant views on the subject, based on how he behaves and talks. I– and apparently, he's of the opposite gender, and when it's – and talking to the opposite gender on such delicate subjects can be very hard (P. 17).

Additionally, adolescents also expressed that they felt embarrassed when they were asked by the school to get the form signed by their parents at home. Participants shared; “They didn't really teach much at my school because usually my parents don't really like to sign [consent] forms, since they're really strict about it” (P.19). Another participant echoed the same experiences; **“**... I think coming from an immigrant family, they don't really like to talk about this kind of stuff, so I had most of my education from school” (P.11). Another shared the same feelings;

It's not culturally relevant…it's not common in my culture, like to do this kind of stuff… Like people who came to university straight from another country, they see all this kind of stuff happening, and they're just not sure about what's going on, and like so it's kind of confusing for them (P. 1).

Some of the adolescents shared that talking about their SRH with their families was a cultural shock for them and for their families.

I feel like that [SRH] would be a huge culture shock… But it might be different if you came to Canada when you're a young child, like in kindergarten. I feel like they wouldn't have as much culture shock and cultural difference. So I feel like that's one of the main issues (P. 3).

#### Structural and financial

In terms of SRH services and resources, the adolescents explained that because of the centers' remote location, they were unable to access them. One participant said, “I was thinking that I wanted to contact a doctor, but I don't know who will drive me, or what will happen, right?” (P. 6). Adolescents added that in addition to their hesitancy for open communication regarding their sexual health, the far location and cost of transportation were another hindrance to accessing sexual health services. “Besides the hesitancy, if there – if I had to go somewhere physically, that's another barrier that would add onto it and strengthen the hesitancy” (P. 9). Another participant added, “there are transportation costs. There's like kind of – you know, again, it's the whole uncomfortable thing that I mentioned before” (P. 3). Likewise, a participant shared the same challenge, “– maybe it should be more affordable, so it doesn't scare them off …, so like if there is something, that's a cheaper alternative, I think they [adolescents] would go for it” (P. 19). Other participants shared the same experience, by sharing ‘that [finance] would be a challenge (P. 14) and that “the transportation of getting there would also be kind of [challenging] especially if you don't have your own car. It's like making time for that” (P. 5). Another participant added that changing their house address affected their access to SRH services and centers. “we used to live closer to the downtown area, or like the heart of the city, and that's kind of where it's located. But we've moved out to like the suburbs, I guess, so it's quite a bit of a drive. But yeah, it's nothing too restrictive”.

### Needs related to SRH

This theme highlights the significance of specific needs reported by adolescents regarding their SRH. The needs as follows include access to SRH services and products and a support system.

#### Access to services and products

Almost all the participants reported that SRH knowledge, services, and products should be accessible to adolescents.

This information should I think compulsory to be available to like adolescents, so they don't have to keep guessing, probably because they don't know, or who haven't been exposed to things like this (P.11).

All participants reported that a digital platform would be convenient and the most preferred method of accessing SRH. This is because physically going out to seek SRH services and knowledge is cumbersome.

It's convenient because… it's online and everyone is able to like access through it…. If you have to ask your family doctor you have to first book an appointment, and then you have to go there and talk to him and then come back, so it's quite time consuming. But when it's like online, you can just call them and then just wait, and then you'll be done in pretty much 30 minutes… it's very convenient (P. 9).

Several participants suggested that the knowledge about SRH services and products should be understandable to adolescents to increase accessibility; “I guess I would say being able to access it, making sure that it's easily readable… legible to younger people, because they have a harder time taking in information” (P. 1).

Various participants reported that holistic SRH knowledge should be accessible and to increase accessibility, sources need to be trustworthy and anonymity and confidentiality should be ensured.

Apparently that's Internet, but also, one must be careful when trusting sources….World Health Organization, or like this organization out there that operates like on a high level, and actually proves their points, like cites their resources, like and those resources are also reliable, like only then, I think, people like can actually trust the sites. Because like to be completely honest, I don't trust like information when it comes from… a journal, like Elle, or stuff like that (P. 23).

I prefer online resources, just because of the anonymity. So if people – like if I feel uncomfortable about the topic, or if I'm too shy or something, I can just search it up. And that feels more comfortable… There's no pressure or anything. Nobody's going to judge you. (P. 20).

Participants stated that schools can also contribute to the accessibility of SRH needs for immigrant adolescents; “I don't know if that's even possible, but if there were check-ups like at school so they wouldn't have to go through their parents and everyone would kind of have to go through it” (P. 16).

Several participants highlighted the accessibility of menstrual products. Specifically, participants described that menstrual products should be cost-free as “access to stuff like pads, tampons, and all of that, is just something that I still think is very important… whether that's having a dispenser in all the like university washrooms” (P. 22).

#### Support system

Many participants reported immigrant adolescents need a person to talk to as a support system.

I think they're expecting just someone to guide them, and so that they don't feel so alone and lost… You go through puberty, and it's just a lot to take in at once. …someone to almost like hold their hand through it would be nice, so like support would be probably one (P. 20).

Participants prefer friends, family, or even a stranger as a support system for them to access services related to SRH. One of the participants stated:

I would say it's like a healthy support system, like with my family… I do appreciate the advice of people that I'm close with…I think like just talking it over with other people, I quite like to keep that connection… especially if they have already had that experience, I tend to trust them a lot (P. 21).

Several participants stated that having a support system was influential in the decisions-making of SRH; “also getting that kind of support within the household to like make independent decisions” (P. 6).

Whoever the support person may be, several participants placed importance on feeling safe and comfortable in their support systems.

Going back to my point about making sure that whoever is there is comfortable is really important, because I feel like that's the only way that the person who's going to be using them will like open up… and like talk about what they need it. So like comfort level… Not necessarily like a instructor, but open space (P. 13).

Several participants spoke about how de-stigmatizing conversations about SRH can increase support systems as immigrant adolescents will approach more strangers or professionals if there was less judgment.

I'd really also like to see… more openness about that kind of stuff, even if it's not just like outright resources. I just think that people should be a lot more comfortable talking about those things, and I feel like that would just be a general resource that I could make use of (P. 21).

Other participants mentioned de-stigmatized behavior needs to be *taught* to create support systems.

I would also say just in general, trying to teach more about how to be open in that way, with your parents, or how you can approach those topics with other people that you might not feel comfortable with, more maturely, and with the partners (P. 13).

### Sources of knowledge

This theme highlights the significance of sources of knowledge related to SRH among adolescents. The major sources of information were the internet, peers, and family as presented below in detail.

#### Internet and media

The participants were asked to share about the source of knowledge on SRH. Most of the participants expressed that the most common source of information was the internet and they mainly rely on YouTube videos to gain knowledge related to their SRH needs.. Another participant added that, “Yeah, basically Google, just Google about it, and the Internet has all the information, so it's a good resource” (P. 4). Similarly, other participants highlighted that the internet was generally useful for obtaining their SRH needs: “It's all Internet, I would say” (P. 6); “I would probably say like the Internet is a big one” (P. 11); “And mainly, like for me, it's the Internet” (P.12); **“**from the Internet, I get it from there, … that I don't know or I'm not aware of, so the Internet is what I look at” (P. 22) Additionally, some participants specifically utilized YouTube as their source of SRH information: “I watched it like a YouTube video. YouTube has been super helpful, like seeing people actually experience stuff and do stuff” (P. 13). “YouTube was very helpful” (P. 16). “I had to do the research all by myself and again, go on YouTube and watch girls' stories, see how they did it” (P. 5).

One participant explained that, “Because I feel like a lot of people are maybe embarrassed to talk about these issues, and so Internet has been really helpful for making decisions about sexual and reproductive health” (P. 6). Another added that, “I just had to Google like what was it. I thought that things were called puberty. Like I didn't even know period was like a term” (P. 2). Likewise, one more adolescent shared that:

So yeah, I had to Google a lot and figure out what it was, and then, yeah, I think that's how I learned. And then asking older friends as well”. those popular YouTubers that talk about periods and everything, so I got some education from there as well (P.15).

#### Friends and peers

The second most common source of information reported by the adolescents was friends. One participant said, “some of my friends were telling me about the IUD” (P. 1). Another added that, “I'd say probably from my friends. I don't think school really covered that” (P. 22). One another participant shared that;

I had a couple of friends that were on birth control, so I asked them about it, and they gave me the pros and cons of it, so that's kind of what helped me make my decision. I'd probably say my friends (P.3).

Some adolescents found that the school was not a helpful source of information for them and they had to mainly rely on their friends for getting information. One participant shared that,

I thought that in schools they seem to dance around the topic of sex, and sexual intercourse in particular. Like they would give us the biological names of what happened, but as kids, we don't know what any of that means. So, you go into it blindly, and then by the time you get to junior high, some of your friends tell you what *really* happens, and that's when you know (P.7).

Similarly, another adolescent added that,

I think I'd say mostly just experiences that I've heard from friends, both negative and good experiences that contributed to a lot of my worldview, and how I approach that subject, as well as how my parents talked about it, or how comfortable they were about those things. Mostly that. Yeah, mostly just friends (P.12).

#### Parents and family

There were very few participants who reported that they learned SRH from their parents or someone from their family. For example, one participant said that, “Well, the information I use is basically from either again, my sister, or the Internet” (P.12). “And like if, you know, or sexual reproductive needs, I would like to go to my family for that, because I do again, like have a lot of women in my family” (P. 11). Another participant also shared the same experience of learning; “I know because of my older siblings, there's like counseling” (P. 3). Those participants who had their elder siblings were helpful for them to learn from them: “I have an older sister, so I knew about things like tampons and pads, and then later I learned about menstrual cups and things like that. So, there's that. I don't know what else, what other stuff to say, to be honest” (P. 1).

### Strategies to improve SRH

Adolescents reported strategies that could help them in improving their SRH. These include making a digital story or platform and maintaining confidentiality.

#### Digital story/platform

Almost all participants reported that a digital strategy is quick, confidential, and convenient. Specifically, a participant explained that “If it's digitally, I can access it quickly. Or if I have a spare minute and I just see it on my phone, I'll check it. And it's quiet too. Yeah, it's convenient” (P.18).

Several participants reported that YouTube is a great digital platform as it showcases shared experiences.

Either go on like a website or go on like YouTube channels. Because sometimes people will make these informative videos about like sexual reproductive health, and then I won't like just trust what they say… I will go through the comment section and see like, “Oh, yeah, I had the same problem, and I did this, and it worked” (P.9).

Participants stated that digital strategies should be trustworthy. Many participants regard resources that are certificated or supported by professionals as trustworthy; “Maybe like a peer-reviewed journal. Or like sometimes WikiHealth when it's like a certified author and you can see their credentials” (P.19).

Numerous participants stated that they would like to have resources or contact information for professional services for various SRH-related issues on a digital platform. A participant reported that resources on violence or emergency help should be on the digital platform. Additionally, the costs of such services should also be disclosed. This participant also mentioned that these links would aid adolescents with mental health issues as well.

Like information about the place, where you could go to, and like how they work. So, if anyone has anxiety or anything like that, they would know ahead of time. As well as like cost … as well as like where you could go to report violence (P.16).

Many participants reported that they would like holistic information on menstruation in the digital platform; “Female reproductive health needs, like discharge and periods, and learning about that” (P.3).

#### Maintaining confidentiality

Almost all participants mentioned that confidentiality is extremely crucial for immigrant adolescents in accessing SRH services, especially confidentiality from their parents; “everything stays confidential, and they won't inform your parents if – if like you don't want them to” (P.1) To increase confidentiality, a participant reported that confidentiality should be legalized with a document. This would make SRH services assuring and comfortable for immigrant adolescents.

A doctor would like to show them some documents that they cannot and will not like – and they are legally obligated to not disclose any information that a teen provides, like with anyone else but the teen… because from what I know from psychology classes, adolescents are like they tend to think conventionally. Like that's actually the name of the stage, when they think based on the standards of society and the law… the most reassuring thing for them would be if a professional is legally obligated to be quiet (P.23).

Another participant added that they would rather seek SRH services from strangers for the sake of confidentiality.

It might be nice to have, I guess, a stranger, who you connected with yourself to help you. Just because in terms of a family doctor, it could be your whole family that is taking treatment from this doctor, so you're scared that your information is not kept private, or I know it probably will be, but a lot of people will have a lot of fear surrounding the subject, so it would be good for them to be able to reach out to a certain person who they *know* they can trust, and who was professional (P. 4).

## Discussion

The current study documents the experiences of immigrant adolescents regarding their SRH concerns and needs in Alberta, Canada. This study highlighted the barriers, information needs, and strategies to improve SRH resources and services from the perspective of immigrant adolescents. Importantly, this study illuminates barriers to accessing resources and services related to SRH health among immigrant adolescents in Canada. Immigrant adolescents in Canada find health centers offering SRH services inaccessible. Despite the vast availability of SRH resources and services, the location and financial cost associated with accessing such services created significant barriers for immigrant adolescents. Our findings reporting the inaccessibility of SRH resources and services are complemented by previous research examining the SRH needs of adolescents (24). The inaccessibility of SRH resources and services highlights the structural barriers embedded within the fabric of the Canadian healthcare system which neglects the distinct needs of vulnerable or diverse populations. Accordingly, these findings speak to the need for future Canadian healthcare services to adapt or adopt SRH resources and services that are inclusive of the unique accessibility needs of immigrant adolescents.

Nonetheless, simply developing SRH resources and services that are practical and financially accessible insufficiently meets the SRH needs among immigrant adolescents in Canada. Past evidence reports that immigrant adolescents are at risk of unintended pregnancies due to poor access to SRH resources and services such as contraceptive methods (9, 12). The current study adds to the phenomenon of inaccessible SRH resources and services by accounting for complexities within cultural and social contexts. From the perspective of immigrant adolescents in Canada, unwanted pregnancies and SRH complications could be avoided by protecting their sexual health such as using birth control methods. However, our participants highlight that immigrant adolescents felt uncomfortable disclosing their SRH preferences or need to their parents given the taboo nature of SRH topics within their socio-cultural contexts. Additionally, this study also documents that although formal SRH information sessions were available to immigrant adolescents in schools, it was difficult to get approval from their parents to attend such sessions due to their family's cultural standards.

As such, participants felt the structural barriers of SRH services are further complicated by means of their parents' cultural expectations. For example, immigrant adolescents reported that they were unable to access general practitioners to receive birth control measures given the requirement of parental consent to obtain such services. These findings are critically important as they highlight the need for future Canadian SRH resources and services to further account for the unique socio-cultural context of this population. The conflicting needs and preferences between adolescents and their parents regarding accessing SRH resources is a critical barrier in meeting the SRH needs of immigrant adolescents. Despite the availability of SRH resources and services, immigrant adolescents felt unprepared to deal with their SRH issues due to cultural conflicts with their parents' conservative attitude toward SRH. Specifically, the participants in this study describe the immense challenges of openly discussing their SRH needs with their parents given the element of shame within their family's cultural practices. Such findings are aligned with previous research on immigrant adolescents' challenges when navigating cultural nuances to meet their SRH needs (12, 15, 23–25). In combination with past literature on this topic, this study demonstrates that SRH services must consider the dimension of socio-cultural expectations specifically for immigrant adolescents. Particularly, these findings suggest that SRH resources and services require addressing structural barriers while being cognizant of conflicting cultural expectations within pre- and-post migration contexts for immigrant adolescents.

In addition to accessing SRH resources and services, socio-cultural contexts also determined immigrant adolescents' level and sources of SRH knowledge. Given the aforementioned barrier to accessing formal SRH resources and services, the majority of immigrant adolescents relied on the internet and media sources for obtaining information related to their SRH needs. Immigrant adolescents reported that digital access to SRH information provides an opportunity to gain knowledge on SRH topics and address their concerns. Specifically, participants described that digital methods protected their anonymity and confidentiality, and were feasible within their socio-cultural contexts. However, participants in this study often relied on informal digital venues such as Youtube and Google search engine. Access to reliable and evidence-based digital SRH information was lacking despite immigrant adolescents' reporting their interest in such resources. These insights suggest that there is a critical opportunity for SRH research and services providers to close the gap in immigrant adolescents unmet SRH needs. Developing and implementing evidence-based digital SRH resources and services may address immigrant adolescents' SRH needs to adequately navigate their distinct structural barriers and socio-cultural contexts.

## Strengths and limitations

This study expands on the existing literature base by offering unique perspectives from adolescent immigrants regarding inclusive strategies to improve sexual and reproductive health (SRH) services. Specifically, developing digital SRH resources and services offers a unique avenue for immigrant adolescents to navigate their distinct structural and socio-cultural barriers. Despite the strengths of our study, three limitations were present. First, our study explored a topic among a population that may deem SRH as sensitive or stigmatized. Accordingly, some of the interviews ranged from 10 to 15 min in duration given the nature of the topic and the possible limited SRH knowledge and experiences of participants. The short interview length may have limited the depth of data obtained. Second, the interviews were conducted virtually. Given the sensitive nature of the topic, participants may have felt uncomfortable discussing interview content within their context (e.g., at home with family members present). The virtual interview context may have limited the types of responses provided to the interviewer, in addition to the interviewer unable to fully capture nonverbal communication. Third, this study did not include a diverse range of genders (majority identifying as girls), which may have influenced the perspectives portrayed in our findings. Future studies may seek to gain perspectives from diverse gender identities.

## Implications of the study

The study results suggest inclusive sexual and reproductive health services for adolescent immigrants in Canada. It is highly recommended that digital SRH resources and services should be offered to adolescent immigrants to meet their unique cultural preferences.

## Recommendations and conclusion

The present study explored the SRH experiences and information needs of immigrant adolescents living in Alberta, Canada. Given the structural and cultural barriers immigrant adolescents face, the future direction in SRH research and services requires the development of inclusive digital methods to access SRH information and resources. Future areas of research inquiry and SRH service provider efforts need to remain cognizant of the unique positionality of immigrant adolescents and explore innovative ways to deliver SRH resources and services that meet the needs of this population.

## Data availability statement

The raw data supporting the conclusions of this article will be made available by the authors upon request.

## Ethics statement

The authors obtained ethical approval from the University of Alberta Ethics Review Board (Pro00097730). Written informed consent from the participants' legal guardian/next of kin was not required to participate in this study in accordance with the national legislation and the institutional requirements.

## Author contributions

SM conceived and co-designed this study. SM, SK, and SL-P conducted interviews. SM, SK, SL-P, and SI led the analysis and drafted the manuscript. All authors commented on all drafts of the article and approved the final draft.

## Funding

This project was supported by Women and Children's Health Research Institute (Grant Number: RES0047403).

## Conflict of interest

The authors declare that the research was conducted in the absence of any commercial or financial relationships that could be construed as a potential conflict of interest.

## Publisher's note

All claims expressed in this article are solely those of the authors and do not necessarily represent those of their affiliated organizations, or those of the publisher, the editors and the reviewers. Any product that may be evaluated in this article, or claim that may be made by its manufacturer, is not guaranteed or endorsed by the publisher.
