# Supplementary material for: Understanding the sexual and reproductive health needs of immigrant adolescents in Canada: A qualitative study
Source: Front Reprod Health. 2022 Jul 22;4:940979. doi: 10.3389/frph.2022.940979 (PMC9580723; doi:10.3389/frph.2022.940979)
Supplement: Supplementary file 1 [file Data_Sheet_1.docx]

**Supplementary File A: Interview Guide for Adolescents**

Initial Open-Ended Questions

1. What do you know about SRH?

2. What do you know about SRHR rights?

3. Tell me briefly about your SRH history?

4. What are some of your needs related to SRH?

5. What are the sources of information that are available for you (or you are aware of) related to your SRH and rights?

a. How did you become aware about those sources?

b. Do you think these information sources are culturally relevant?

c. Do you think these information sources are trustworthy?

6. What information did you use to help make your decision related to SRH?

7. What was most helpful to you in making your decision related to SRH?

8. What are the challenges you have faced in receiving SRH information?

9. Have you ever used SRH services?

a. Have you heard of any SRH services in the city?

b. How involved do you perceive your parents to be involved in decisions to use these services or know about them?

10. How was your experience overall thoughts and feelings about accessing SRH services?

11. What is easy/hard about accessing SRH services?

a. What were barriers in accessing SRH services?

12. Are you familiar with SRH mobile apps or websites, etc. you can use to receive SRH information?

a. If yes, what are those resources and how helpful those resources for you?

b. If not, would you be interested in knowing?

13. Do you recommend developing a digital strategy (e.g., Mobile app, website) to help you access SRH information easily?

a. If you did recommend it, what types of information would you like to see on the digital platform?

Ending Questions

1. Have your thoughts or feelings about SRH changed over time?

2. What do you think adolescents are expecting or prefer from SRH service providers and services?

3. Is there anything that you might not have thought about before that occurred to you during this interview?

4. Is there anything else you think I should know to better understand adolescents SRH information needs?
