# Supplementary material for: Understanding the sexual and reproductive health needs of immigrant adolescents in Canada: A qualitative study
Source: Front Reprod Health. 2022 Jul 22;4:940979. doi: 10.3389/frph.2022.940979 (PMC9580723; doi:10.3389/frph.2022.940979)
Supplement: Supplementary file 2 [file Data_Sheet_2.docx]

**Supplementary File B: Demographic Characteristics of the Participants’ Parents**

| ***Characteristics*** | ***Number (f)*** | ***Percentages (%)*** |
| --- | --- | --- |
| **Father’s Country of Birth**  India  Philippine  Bangladesh  China  Nigeria  Nepal  Columbia  Venezuela  Canada  Malaysia  Kazakhstan | 10  2  2  1  2  1  1  1  1  1  1 | 43%  8.7%  8.7%  4.4%  8.7%  4.4%  4.4%  4.4%  4.4%  4.4%  4.4% |
| **Mother’s Country of Birth**  India  Philippine  Bangladesh  China  Nigeria  Nepal  Columbia  Venezuela  Canada  Indonesia  Kazakhstan | 10  2  2  1  2  1  1  1  1  1  1 | 43%  8.7%  8.7%  4.4%  8.7%  4.4%  4.4%  4.4%  4.4%  4.4%  4.4% |
|  |  |  |
